# The emerging role of FTY720 as a sphingosine 1‐phosphate analog for the treatment of ischemic stroke: The cellular and molecular mechanisms

**DOI:** 10.1002/brb3.2179

**Published:** 2021-05-10

**Authors:** Maryam Naseh, Jafar Vatanparast, Ali Rafati, Mahnaz Bayat, Masoud Haghani

**Affiliations:** ^1^ Histomorphometry and Stereology Research Centre Shiraz University of Medical Sciences Shiraz Iran; ^2^ Department of Biology School of Science Shiraz University Shiraz Iran; ^3^ Department of Physiology Shiraz University of Medical Sciences Shiraz Iran; ^4^ Clinical Neurology Research Center Shiraz University of Medical Sciences Shiraz Iran

**Keywords:** FTY720, ischemic stroke, neuroprotection, sphingosine 1‐phosphate (S1P) receptor

## Abstract

Finding novel and effective drugs for the treatment of ischemic stroke is warranted because there is not a definitive treatment for this prevalent disease. Due to the relevance between the sphingosine 1‐phosphate (S1P) receptor and several neurological diseases including ischemic stroke, it seems that fingolimod (FTY720), as an agonist of S1P receptor, can be a useful therapeutic strategy in these patients. FTY720 is the first oral drug approved by the US food and drug administration for the treatment of multiple sclerosis. Three important mechanisms for neuroprotective effects of FTY720 have been described. First, the functional antagonistic mechanism that is associated with lymphopenia and reduced lymphocytic inflammation. This effect results from the down‐regulation and degradation of lymphocytes' S1P receptors, which inhibits lymph node lymphocytes from entering the bloodstream. Second, a functional agonistic activity that is mediated through direct effects via targeting S1P receptors on the membrane of various cells including neurons, microglia, oligodendrocytes, astrocytes, and endothelial cells of blood vessels in the central nervous system (CNS), and the third, receptor‐independent mechanisms that are displayed by binding to specific cellular proteins that modulate intracellular signaling pathways or affect epigenetic transcriptions. Therefore, we review these mechanisms in more detail and describe the animal model and in clinical trial studies that support these three mechanisms for the neuroprotective action of FTY720 in ischemic stroke.

## Graphical abstract

Three important mechanisms for neuroprotective effects of FTY720 have been described. Functional antagonistic, functional agonistic and receptor‐independent mechanisms. Here, we review these mechanisms in more details and describe animal model and in clinical trial studies

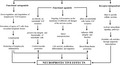

## 1 INTRODUCTION

### 1.1 Stroke pathophysiology

Stroke is the second most common cause of death worldwide and the leading cause of long‐term disabilities (Campbell et al., 2019). The main types of stroke are ischemic stroke, hemorrhagic stroke, and transient ischemic attack. The ischemic strokes are the most common, accounting for about 87% of all strokes. It occurs when a vessel supplying blood to the brain is obstructed resulting in thrombotic, embolic agent, or systemic hypoperfusion (Musuka et al., 2015; Price et al., 2018). When brain ischemia occurs, triggering deleterious cascades in the brain that mediate pathological changes including excitotoxicity, acidotoxicity, and ionic imbalance, peri‐infarct depolarization, oxidative/nitrative stress, inflammation, and apoptosis (Doyle et al., 2008). Each of the pathophysiological processes occurs in a distinct period of minutes‐to‐days following the onset of symptoms.

In the brain ischemia, the central region of brain tissue is infarcted rapidly and is termed the core of the infarct, and the region around the core is known as the ischemic penumbra. The molecular mechanisms of cellular injury and death are different in these two regions. In the core region, where blood flow is most severely restricted cellular death is often accompanied by excitotoxicity and necrosis within minutes, while in the ischemic penumbra, where collateral blood flow can reduce the effects of severe hypoperfusion, the fate of neural cells is determined by the degree and duration of ischemia. In the penumbra area, cell death occurs at a slower rate than central region cell death. Apoptosis is the main target mechanism of cell death in penumbra that occurs less rapidly and provides the therapeutic opportunity (Gonzalez et al., 2006). Moreover, brain injury following stroke results from the infiltration of inflammatory cells of both innate and adaptive immune systems, which can accompany disruption of blood‐brain‐barrier (BBB), neuronal loss, microglial activation, and formation of the glial scar. These consequences lead to more neurological deficits (Khoshnam et al., 2017).

The only approved therapy that can be used for patients beyond the narrow time window is activating the thrombolysis with the infusion of recombinant tissue‐type plasminogen activator (rt‐PA) (Cheng & Kim, 2015). Due to a narrow time window and the high risk of hemorrhage, this treatment is not a safe therapy. Therefore, identifying novel and effective targets for the treatment of these patients is urgently needed. The FTY720 is a partial agonist of the sphingosine‐1‐phosphate (S1P) receptor with immunomodulatory properties. In 2010, it was introduced as a FDA‐approved drug for the treatment of multiple sclerosis (MS) (Doggrell, 2010; Kappos et al., 2010). Concerning the results from the clinical trials (Fu et al., 2014; Zhu et al., 2015), and the role of the immune system in the pathophysiology of ischemic stroke (Chamorro et al., 2012; Iadecola & Anrather, 2011; Planas et al., 2012; Yilmaz et al., 2006), it seems that FTY720 may provide a useful therapeutic strategy in stroke patients (Lucaciu et al., 2020). Therefore, this study reviews the purpose of neuroprotective mechanisms of FTY720, aiming to provide an overview and its implications on ischemic stroke.

### 1.2 Production and metabolism of sphingolipids

Sphingolipids are among the main components of the plasma membrane of eukaryotic cells and some prokaryotes, which are ubiquitous in the brain and act as second messengers that regulate cell programs (Huwiler & Pfeilschifter, 2008). The conversion of serine and palmitoyl coenzyme A (CoA) into ceramide is the beginning step for the synthesis of sphingolipids (Kawabori et al., 2013). Ceramide is densely present in the cell membrane and is a major component of the phospholipid bilayer (Bikman & Summers, 2011). It is the precursor for bioactive lipid mediators like sphingosine and sphingosine 1‐phosphate (S1P). S1P is a bioactive metabolic product of sphingolipids generated by sphingosine kinases (Sphk) and can act as an intracellular second messenger and can also be sent out of the cell, displaying as an extracellular signaling ligand and activates five specific S1P families of G protein‐coupled receptors (S1P1‐5) (Singh & Hall, 2008). These receptors activate signaling pathways that control cell survival, proliferation, differentiation, migration, adhesion, cytoskeletal organization (Chun et al., 2002; Hannun & Obeid, 2008). It has been shown that bioactive species of sphingolipids are involved in numerous cellular signaling cascades and could have different biological effects. Ceramide and sphingosine, as intracellular second messengers, activate apoptotic pathways. On the other hand, S1P induces activation of anti‐apoptotic pathways and antagonizes the effects of ceramide and sphingosine. Thus, the physiological balance between ceramide, sphingosine, and S1P controls the cell fate (Cuvillier et al., 1996; Morad & Cabot, 2013; Proia & Hla, 2015). Sphingosine is phosphorylated to produce S1P by sphingosine kinases (SphKs) and S1P can be dephosphorylated back to sphingosine by ER‐resident enzyme that specifically dephosphorylates S1P. The rate of production and elimination of S1P is precisely regulated by SphKs and S1P lyase.

The conversion of sphingosine into S1P is catalyzed by SphKs as the rate‐limiting enzymes in the process of endogenous S1P generation. There are two isoforms of SphKs 1 and 2 (Sphk1 and Sphk2). In addition to the synthesis of S1P, SphKs also catalyzes the phosphorylation of FTY720 (Bryan et al., 2008). Unlike many other organs, SphK2 is the principal isoform of SphKs in the brain. It is highly expressed and shows strong activity in neuronal and glial cells, and also in cerebral microvascular endothelial cells (Billich et al., 2003; Blondeau et al., 2007). It has been suggested that S1P via vascular endothelial cells regulate calcium mobility, migration, proliferation, survival, angiogenesis, and permeability of vasculatures. (Bryan et al., 2008; Kluk & Hla, 2002; Saba & Hla, 2004).

### 1.3 FTY720 as a sphingosine 1‐phosphate analog

FTY720 was first described in 1995 as a synthetic analog of sphingosine derived from myriocin, a toxin of the fungus *Isaria sinclairii* (Adachi et al., 1995). In 2010, the FTY720 was approved as the first oral drug for the treatment of multiple sclerosis by the United States Food and Drug Administration (Strader et al., 2011). It can enter the central nervous system and interact with S1P receptors (Meno‐Tetang et al., 2006). It is phosphorylated to FTY720‐phosphate (FTY720‐p) predominantly by SphK2, which is highly expressed in the brain and brain microvasculature (Wacker et al., 2009). FTY720‐pacts as an agonist at S1P1, S1P3, S1P4, and S1P5 receptors (Brinkmann et al., 2002) and especially acts as an agonist for neuronal S1P1. It inhibits the inflammatory responses in the brain by acting on S1PRs, principally S1PR1, which controls lymphocyte trafficking. FTY720 also directly affects other neuronal and immune cells (Mehling et al., 2011). It has been also shown that FTY720 is involved in multiple processes including cell survival, proliferation, differentiation, and migration (Miron et al., 2008).

**FIGURE 1:**
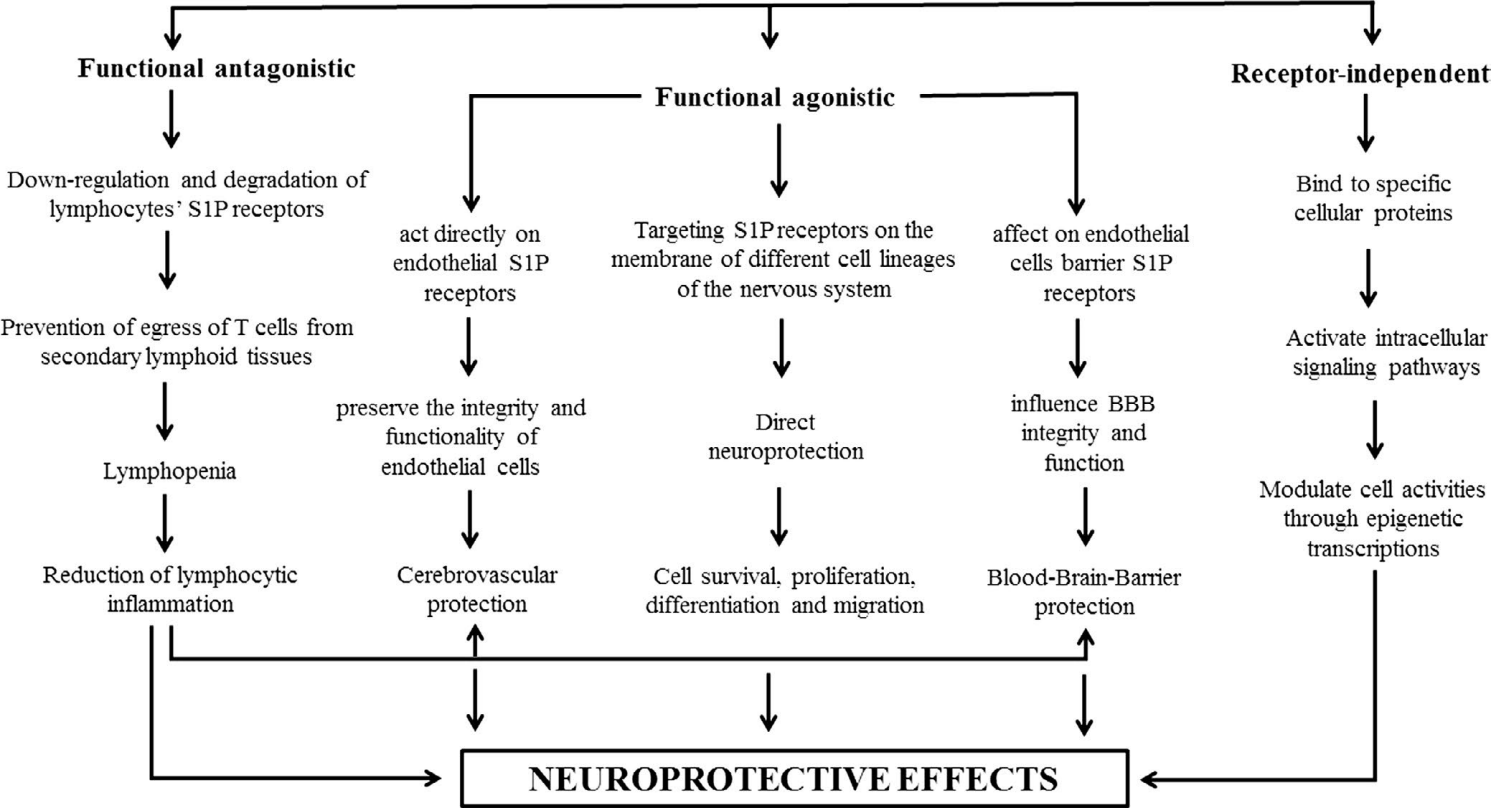
Schematic representation of neuroprotective effects of FTY720

Three mechanisms have been suggested for the neuroprotective effects of FTY720 (Wang et al., 2020) (Figure 1). First, the functional antagonistic mechanism that induces immunomodulatory actions through lymphopenia (Chiba et al., 1998; Matloubian et al., 2004) Second, a functional agonistic activity that is related to its pro‐survival and anti‐inflammatory effects (Sanchez et al., 2003; Xin et al., 2007), and the third, receptor‐independent mechanisms that are displayed by binding to specific cellular proteins that modulate intracellular signaling pathways or affect epigenetic transcriptions (Gardner et al., 2016; Hait et al., 2014; Segura‐Ulate et al., 2017).

## 2 THE NEUROPROTECTION MECHANISMS OF FTY720

### 2.1 Functional antagonist mechanism

The primary and most prominent mechanism of the action of FTY720, which is known as the functional antagonistic mechanism, is mediated by its lymphocyte homing action. Phosphorylated FTY720 binds to lymphocyte S1P1 receptors and causes the internalization and degradation of the receptor (Figure 1). It leads to endured desensitization of the S1P1 signaling cascade and reduces the response of T cell to S1P1 receptors (Matloubian et al., 2004), causing prevention of egress of T cells from secondary lymphoid tissues. It also reversibly redistributes T and B cells from the bloodstream to secondary lymphoid organs like peripheral and mesenteric lymph nodes, and subsequently, induces lymphopenia and reduces lymphocytic inflammation (Chiba et al., 1998; Matloubian et al., 2004). This effect of FTY720 on S1P1 is unique and different from the endogenous ligand of S1P. FTY720 also reduces the infiltration of lymphocytes in the cerebral blood vessels after ischemic stroke (Kraft et al., 2013). Lymphocytes are important players in the pathogenesis and tissue injury of stroke (Yilmaz et al., 2006). They can work as modulators of leukocyte and platelet adhesion following ischemic stroke (Shichita et al., 2009). Activated T lymphocytes appear as early as 24 hr after reperfusion in ischemic brain tissue and they produce inflammatory cytokines such as IL‐17 and IFN‐γ in the site of injury. It has been demonstrated that CD4^+^ helper, CD8^+^ cytotoxic, and γδT cells play damaging roles in experimental stroke (Shichita et al., 2009; Yilmaz et al., 2006). The deficiency of CD4^+^ helper/CD8^+^ cytotoxic T cells led to decreased number of adherent leukocytes and lymphocytes, and causes smaller ischemic infarct sizes and amelioration of neurological outcomes following tMCAO in mice (Yilmaz et al., 2006). Induction of lymphopenia and concomitant reduction of microvascular thrombosis by FTY720 can decrease stroke size and improve functional outcome in the model of tMCAO. This protective effect of FTY720 is lost in lymphocyte‐deficient *Rag1−/−* mice (Kraft et al., 2013). Another study also showed that lymphopenia could be the essential contributor for the stroke‐protective properties of FTY720 in the mice model of MCAO (Czech et al., 2009). Treatment with FTY720 decreases the numbers of infiltrating neutrophils, activated microglia/macrophages, and T lymphocytes in the ischemic lesion. It could reduce lesion size and improve neurological function after experimental stroke in mice (Czech et al., 2009). It has been proposed that FTY720 could be a therapeutic agent for mitigating the inflammatory events through blockage of the T lymphocytes, including γδT lymphocytes, infiltration into the brain that amplify the initial damage in cerebral ischemia (Shichita et al., 2009). It has been also suggested that neuroprotection effects of FTY720 are accompanied by decreased inflammation and possibly vasculo‐protection rather than direct effects on neurons after stroke (Wei et al., 2011). Decreased inflammation and vasculo‐protection could be obtained via reducing the number of activated neutrophils and microglia/macrophages and ICAM‐1 positive blood vessels (Wei et al., 2011) (Tables 1 and 2).

**TABLE 1:** Comparison of the neuroprotective effects of FTY720 in different methods of ischemic stroke in experimental and clinical studies

Author, year	Stroke models	Different animal species	Different doses of FTY720	Method of administration	Results	Reference
Brait VH, 2016	45 min MCAO and reperfusion	Mice	1 mg/kg	Intraperitoneal (i.p.)	FTY720 reduced infarct volume	Brait et al. (2016)
Salas‐Perdomo, 2019	45 min MCAO and reperfusion	Mice	1 mg/kg	i.p.	Fingolimod attenuated the extent of bleeding and improved the neurological score	Salas‐Perdomo et al. (2019)
Nazari M, 2016	60 min MCAO and reperfusion	Male Sprague‐Dawley rats	0.5 mg/kg	i.p.	FTY720 improved the infarct volume and memory performance	Nazari et al. (2016)
Czech B, 2009	90 min MCAO and reperfusion	C57Bl/6 mice	1 mg/kg	i.p.	FTY720 reduced lesion size and improved neurological function	Czech et al. (2009)
Wacker BK, 2009	60 min tMCAO and 24 hr of reperfusion	Adult male Swiss‐Webster ND4 mice	0.24 or 1 mg/kg	i.p	FTY720 dose‐dependently decreased infarct size and neurological deficit	Wacker et al. (2009)
Wei Y, 2011	90 min tMCAO and reperfusion/ Permanent MCAO	Rat/ C57BL/6 male mice	0.5 or 1 mg/kg	i.p.	FTY720 reduced infarct size and neurological deficit	Wei et al. (2011)
Hasegawa Y, 2013	120 min tMCAO and reperfusion	Sprague‐Dawley rats	0.25 mg/kg	i.p.	FTY720 improved infarction size and neurological function	Hasegawa et al. (2013)
Hasegawa Y, 2010	120 min tMCAO and reperfusion	Sprague‐Dawley rats	0.25, 1 mg/kg	i.p.	FTY720 significantly reduced infarct volume and improved neurological score	Hasegawa et al. (2010)
Kraft P, 2013	60 or 90 min tMCAO and reperfusion	C57Bl/6 mice	1 mg/kg	i.p.	FTY720 significantly reduced stroke size and improved functional outcome	Kraft et al. (2013)
Moon E, 2015	60 or 90 min tMCAO and reperfusion	ICR male mice	3 mg/kg	i.p.	FTY720 reduced ischemic brain damage	Moon et al. (2015)
Pfeilschifter,W, 2011	120 min tMCAO and reperfusion	C57Bl/6 mice	1 mg/kg	i.p.	FTY720 was effective in improvement of brain injury	Pfeilschifter et al. (2011)
Shichita, 2009	60 min MCAO and reperfusion	C57/BL/6 male mice	1 mg/kg	Intravenous (i.v.)	FTY720 reduced I/R‐induced brain damage reduced infarct volume	Shichita et al. (2009)
Liesz A, 2011	60 min Permanent MCAO	C57BL/6 male mice	1 mg/kg	i.p.	FTY720 did not reduce infarct volumes and improve behavioral dysfunction	Liesz et al. (2011)
Qin Ch, 2017	Hypoperfusion‐induced White matter ischemic injury	C57Bl/6 mice	0.3 mg/kg	i.p.	FTY720 has neuroprotective effect in acute ischemic stroke	Qin et al. (2017)
Campos F, 2013	80 min thromboembolic occlusion and reperfusion	C57BL/6 male mice	0.5 mg/kg	i.p.	Fingolimod attenuated the neurological deficit and infarct volume	Campos et al. (2013)
Brunkhorst R,2013	15 min photothrombosis	C57BL/6 male mice	1 mg/kg	i.p.	FTY720 has a positive impact on long‐term functional outcome	Brunkhorst et al. (2013)
Li X,2017	20 min thromboembolic occlusion	C57/BL/6 male mice	0.5, 1, or 2 mg/kg	i.p.	FTY720, attenuated cerebral infarction, neuronal apoptosis, and improved neurological deficits	Li et al. (2017)
Shang K, 2020	15 min thromboembolic occlusion	C57/BL/6 male mice	0.3 mg/kg	i.p.	FTY720 reduced neuronal loss and improved motor function	Shang et al. (2020)
Yang D, 2014	90 min hypoxic–ischemic (HI)	Wistar rat pups	0.3 or 1 μg/g,	i.p.	FTY720 prevent inflammation‐sensitized hypoxic–ischemic brain injury in newborns	Yang et al. (2014)
Fu Y, 2014	Patients with acute ischemic stroke		0.5 mg/day	Orally for 3 consecutive days	FTY720 decreased lesion size, microvascular permeability and attenuated neurological deficits	Fu et al. (2014)
Zhu Z, 2015	Patients with acute ischemic stroke		0.5 mg/day	Orally for 3 consecutive days	FTY720 plus alteplase reduced infarction size, hemorrhage volume and improved clinical outcomes	Zhu et al., (2015)

**TABLE 2:** The simplified summary of proposed neuroprotective mechanisms of FTY720 at ischemic stroke

FTY720 action		Mechanism(s)	Reference(s)
Functional antagonist		Lymphopenia	Chiba et al. (1998); Matloubian et al. (2004); Czech et al. (2009); Kraft et al. (2013); Shichita et al. (2009); Wei et al. (2011)
		↓ ICAM‐1	Li, Shi, et al., (2020); Wei et al. (2011)
Functional Agonist	Cerebrovascular protection	Direct action on endothelial S1P receptors	Li, He, et al., (2020); Wei et al. (2011)
		↓ ICAM‐1	Li, He, et al., (2020); Wei et al. (2011)
	BBB protection	↓ Endothelial apoptosis	Limaye et al. (2005)
		↑ Protein ZO‐1	Lee et al. (2006)
		↑ Cadherins	Prager et al. (2015)
		↓ Trafficking of immune cells	Campos et al. (2013); Liesz et al. (2011); Rolland et al. (2013); Wei et al. (2011)
	Direct neuroprotection	PI3K/Akt/FOXO3a pathway	Safarian et al. (2015)
		ERK/bcl‐2 pathway	Hasegawa et al. (2010)
		↓ Neuronal autophagy	Li et al. (2017)
		↑ Neurogenesis (mediated by BDNF)	Deogracias et al. (2012); Fukumoto et al. (2014)
		↓Excitotoxic cell death	(76)
		↓ Astrogliosis	Brunkhorst et al. (2013)
		↑ Angiogenesis through the microglial polarization state toward M2 phenotype	Shang et al. (2020)
		↓ Demyelination by skewing microglia toward M2 polarization (STAT3 pathway)	Qin et al. (2017)
		↑ Myelination potential of oligodendrocyte	Miron, Hall, et al., (2008); Miron, Jung, et al., (2008); Miron et al. (2010)
Receptor‐Independent		↑ Acetylation of histone ↑ Neurotrophic factor generation ↓ T cell activation	Gardner et al. (2016); Hait et al. (2009); Hait et al. (2014); Pfeilschifter et al. (2011); Segura‐Ulate et al. (2017)

### 2.2 Functional agonist mechanism

Another mechanism that is proposed for FTY720 is its direct neuroprotective effects.

S1P1 is widely expressed in the brain and neurons express mainly S1P1 and S1P3 (Dev et al., 2008). Therefore, FTY720 could have direct actions on neurons (Shabani et al., 2018; Shabani et al., 2019). It also provides vascular protection in the brain mainly through its immunomodulatory actions. There are also several reports that S1P may act as a trigger of protection of both neuronal and cerebral vessels via activation of signaling molecules such as Akt and eNOS (Morales‐Ruiz et al., 2001; Puisieux et al., 2000; Zhang et al., 2007) (Figure 1).

#### 2.2.1 Cerebrovascular protection

Cerebral ischemia activates leukocyte adhesion and initiation of inflammation in the brain and this is mediated by the interaction of leukocytes' β2‐integrins with ICAM‐1 on cerebral endothelial cells (Figure 1). Leukocyte accumulation causes the generation of oxygen free radicals, the release of cytotoxic enzymes, cytokines, and chemoattractants. Besides, it occludes the microvasculature and obstructs blood flow, which is called the no‐reflow phenomenon. Formation of the leukocyte–platelet complexes following brain ischemia could also cause further damage by plugging the microvasculature (Danton & Dietrich, 2003; Kawabori & Yenari, 2015). The generation of free radicals by activated neutrophils can also damage the microvascular endothelium and induce a detrimental rise in intracranial pressure and edema. It has been shown that FTY720 could ameliorate brain ischemia‐induced edema using two mechanisms: first, by decreasing ICAM‐1 expression and neutrophil activation; second, by direct action on endothelial S1P receptors. After being phosphorylated by sphingosine kinase 2, FTY720 acts directly on endothelial S1P receptors to preserve the integrity and functionality of endothelial cells and to decrease vascular permeability and progression of vascular inflammation (Li et al., 2020; Wei et al., 2011) (Tables 1 and 2).

#### 2.2.2 Recovery of blood‐brain‐barrier disruption

The blood‐brain barrier (BBB) is a multicellular vascular structure that is composed of endothelial cells, basement membrane, astrocytes, and pericytes (Serlin et al., 2015). The BBB separates the central nervous system from the peripheral blood circulation. It plays a crucial role in the maintenance of ionic balance required for neurotransmission and prevention of the excessive entry of immune cells (Ransohoff & Engelhardt, 2012). It has been reported that ischemic stroke disturbs the physical integrity of the blood‐brain barrier and increases permeability, which can allow uncontrolled brain entrance and causes cytotoxic effects (Ballabh et al., 2004). FTY720 could readily cross the BBB and lead to neuroprotective effects (Gillingwater, 2012; Miron, Schubart, et al., 2008).

Since S1P receptors are expressed on endothelial cells, FTY720 can affect endothelial cell barrier mostly through S1P1 receptors to influence BBB integrity and function (Camp et al., 2009). It has been demonstrated that S1P reduces leukocyte adhesion by altering endothelial adhesion molecule expression and can protect the vasculature (Awad et al., 2006), and prevents endothelial apoptosis via Bcl‐2 activation (Limaye et al., 2005). S1P was also shown to enhance BBB endothelial barrier integrity by stimulating tight‐junction‐associated protein ZO‐1 at cellular junctions (Lee et al., 2006). In addition, FTY720 could also stimulate BBB endothelial cells to recruit adherens junction proteins such as cadherins, leading to the maintenance of endothelial barrier properties such as vascular permeability and restriction of neutrophil infiltration (Prager et al., 2015). Some researchers have found that FTY720 can maintain BBB structure and function in ischemic stroke. Wang et al. reported that FTY720 via S1P1 receptor could protect BBB integrity by inhibiting the redistribution of lamellipodia‐located tight and adherens junctions, ZO‐1 and VE‐cadherin into the cytoplasm, as well as decreasing inflammation in the subacute phase of the stroke model (Wang, Higashikawa, et al., 2020). It could also reduce transendothelial trafficking of immune cells in cerebral ischemia and intracranial hemorrhage (Campos et al., 2013; Liesz et al., 2011; Rolland et al., 2013; Wei et al., 2011). But, some findings were discrepant to these studies and showed FTY720 cannot relieve BBB breakdown or local inflammation, at least during the acute phase of ischemic insult (Kraft et al., 2013). Although acute FTY720 treatment could reduce the infiltration of MPO positive inflammatory cells and TNF‐α gene expression at 24 hr after tMCAO in diabetic mice, it significantly exacerbated BBB leakage and edema (Li, He, et al., 2020). The exact reasons for these opposite findings are unclear, but differences in the stroke models, animal species, and different doses of FTY720 that used may contribute to different results in terms of BBB stabilization and immune cell transmigration (Hasegawa et al., 2010; Wei et al., 2011) (Tables 1 and 2).

#### 2.2.3 The protective effects of FTY720 on different cell lineages of the nervous system; direct neuroprotection

The different cell lineages of the nervous system such as neurons, astrocytes, microglia, and oligodendrocytes express S1P receptors (Hasegawa et al., 2010; Liesz et al., 2011; Soliven et al., 2011). All types of S1P receptors are expressed on neuronal and glial cells of the CNS, except S1P4 (Chun et al., 2000). The S1P4 receptor is thought to be restricted to the hemapoietic system and is not expressed in the brain (Malchinkhuu et al., 2003). S1P receptors apparently contribute to neurite outgrowth and neurogenesis (Harada et al., 2004; MacLennan et al., 2000). Therefore, it is likely to assume that FTY720 can promote this process. FTY720 may exert its protective actions through direct interaction with neuronal S1P1 and S1P3 receptors, which activates the production of anti‐apoptotic factors and increases resilience against ischemic injuries. There is also in vivo stroke model evidence that confirms FTY720 can protect neuronal cells via the S1P1 receptor in response to ischemic stroke (Hasegawa et al., ,^2010^, ^2013^). One mechanism proposed for direct neuroprotection of FTY720 is the S1P/Phosphatidylinositol‐3‐Kinase (PI3K)/Akt/FOXO3a axis, which has an essential role in mediating oxidative stress‐induced cell death. It has been suggested that FTY720 prevents activation of FOXO3a via phosphorylation of Akt, and suppression of FOXO3a may contribute to neuronal survival in ischemic insults (Safarian et al., 2015). It could also provide neuroprotection through the ERK/BCL‐2 pathway. FTY720 induces persistent ERK phosphorylation and enhances the levels of the anti‐apoptotic protein Bcl‐2, protecting neurons from apoptosis in ischemic condition (Hasegawa et al., 2010).

FTY720 could effectively alleviate ischemic brain damage by suppressing the neuronal autophagy via reduction of the autophagosome proteins, microtubule‐associated protein 1 light chain 3 (LC‐3‐II) and Beclin1, and upregulation of the mammalian target of rapamycin (mTOR) signaling pathway (Li et al., 2017). Neuronal autophagy seems to be associated with the loss of neuronal cells and has a vital role in the development of ischemic stroke (Li et al., 2017). It has been also shown that FTY720 exerts direct effects on neurons, astrocytes, microglia, and oligodendrocytes which are all affected following ischemic stroke (Hunter et al., 2016). Meanwhile, some researchers believe that the beneficial effects of FTY720 are not related to direct effects on neurons after stroke (Wei et al., 2011). Some animal evidence supported the action of FTY720 in improving neuronal function and neuroprotection in other neurological diseases. They suggested that the effects of FTY720 on neurogenesis could be mediated by BDNF (Deogracias et al., 2012; Fukumoto et al., 2014). It has been suggested that FTY720 also protects neurons against excitotoxic cell death (Di Menna et al., 2013).

Astrocytes actively contribute to supportive, metabolic, and immunoregulatory functions in the brain (Markiewicz & Lukomska, 2006). Ischemic stroke causes an extensive glial response as reactive gliosis. Reactive gliosis, in turn, leads to the formation of a glial scar at the site of injury. Activated astrocytes invade and proliferate at the ischemic lesion to form the glial scar (Huang et al., 2014). The responses of glial cells to ischemic stroke have been studied in the animal models using photothrombosis and focal transient or permanent middle cerebral artery occlusion (Lively et al., 2011; Morioka et al., 1993; Nowicka et al., 2008). Furthermore, glial scar formation appears in the human brain after ischemic stroke (Huang et al., 2014). Phosphorylated FTY720 has an anti‐inflammatory effect on human astrocytes by decrement of pro‐inflammatory cytokines secretion. It has been also seen that reactive astrocytes strongly enhance the expression of S1P1 and S1P3 in pro‐inflammatory conditions (Van Doorn et al., 2010). It seems that S1P receptors play important roles in the proliferation and migration of astrocytes in the brain (Malchinkhuu et al., 2003; Mullershausen et al., 2007). The expression of pro‐inflammatory and pro‐apoptotic proteins could be reduced in astrocytes in response to FTY720, as well as increased neurotrophic factors, such as BDNF and nerve growth factor (Hoffmann et al., 2015). The astrocytes could be a cellular target of FTY720 action. It has been considered that FTY720 contributes to in reducing the activation of astrocytes in several neurological diseases including ischemic stroke (Brunkhorst et al., 2013). The improved functional outcome after FTY720 treatment in the convalescence period is mediated through inhibited astrogliosis and an increased expression of neurotrophic factors in photothrombotic stroke (Brunkhorst et al., 2013).

Microglial cells comprise the resident immune cells of the brain. They are the first and main form of active immune defense in the CNS and contribute to modulating the inflammatory responses (Ginhoux et al., 2013). It has been reported that microglia undergo morphological changes and migrate to the sites of injury after brain damage (Kimura et al., 2007). Activated microglia exhibit two distinct functional phenotypes. These are distinguished as the M1‐(classical/pro‐inflammatory activation) and M2‐(alternative/anti‐inflammatory activation) types. The balance between pro‐inflammatory and anti‐inflammatory activated microglial phenotypes indicates the development of central nervous system diseases (Jha et al., 2016; Qin et al., 2019). The M1 phenotype is primarily associated with pro‐inflammatory signaling. Microglia are polarized predominantly to a M1 phenotype following brain injury and produces cytokines, ROS, and reactive nitrogen species (Liu et al., 2019; Perego et al., 2013). In contrast, the M2 phenotype appears to contribute to anti‐inflammatory responses as well as recovery and repair. Cytokines, including IL‐4, IL‐10, IL‐13, IGF‐1, and transforming growth factor (TGF)‐β are associated with the M2 phenotype (Cherry et al., 2014; David & Kroner, 2011; Liu et al., 2019; Perego et al., 2013).

Microglial activation is essentially involved in the pathology of ischemic stroke especially in the peri‐infarct region. It has been reported that microglia serve as the first line of defense to the site of ischemic injury and could respond earliest following an insult (Morioka et al., 1991; Weinstein et al., 2010). Microglial accumulation is also one of the earliest cellular reactions in cerebral ischemia (Gelderblom et al., 2009). Three of five S1P receptor subtypes (S1P_1_ (Gaire, Lee, et al., 2018), S1P_2_ (Sapkota et al., 2019), and S1P_3_ (Gaire et al., 2018) are involved in cerebral ischemia, which can affect the polarization of M1 microglial activation in the ischemic brain. These S1P receptor subtypes can activate effector pathways in the ischemic brain in a slightly different way. S1P_1_ activates all 3 MAPKs (ERK1/2, p38, and JNK) (Gaire, Lee, et al., 2018). S1P_2_ activates ERK1/2 and JNK (Sapkota et al., 2019), while S1P_3_ activates ERK1/2 and p38 MAPK (Gaire, Song, et al., 2018). Conversely, it has been demonstrated that FTY720 treatment exerts neuroprotection and promoted angiogenesis through the microglial polarization state toward an M2 phenotype in mice models of acute cortical ischemic stroke (Shang et al., 2020). FTY720 ameliorated demyelination by skewing microglia toward M2 polarization via STAT3 pathway in the ischemic white matter damage (Qin et al., 2017). It should be mentioned that angiogenesis in the ischemic boundary zone is one of the fundamental mechanisms of FTY720's neuroprotection. FTY720 could lead to the enhanced angiogenesis and improvements in the microenvironment of vessel growth via transformation of the microglial phenotype toward an anti‐inflammatory state (Shang et al., 2020).

FTY720 could lead to the enhanced angiogenesis and improvements in the microenvironment of vessel growth via transformation of the microglial phenotype toward an anti‐inflammatory state (Shang et al., 2020). Oligodendrocytes are the myelin‐producing cells that insulate axons to support neural transmission in the CNS. Owing to the expression of S1P1,3,5 in oligodendrocyte lineage cells, they are potential targets for FTY720 (Dev et al., 2008). Considering the expression of S1P5 is undetectable or low in neurons (Harada et al., 2004) and astrocytes (Rao et al., 2003, 2004) but its expression level in oligodendrocyte is high, it is suggested that it may play an important role in regulating oligodendrocytes function (Dev et al., 2008). S1P receptors are thought to have critical roles in oligodendrocyte differentiation and cell survival (Jaillard et al., 2005; Saini et al., 2005). The reduction of demyelination and enhancement of remyelination after FTY720 treatment suggest that increasing the myelinating potential of oligodendrocyte may potentially promote tissue repair and functional recovery in neurological diseases (Miron et al., 2008, 2010; Miron, Jung, et al., 2008) (Table 2).

### 2.3 Receptor‐independent mechanisms

The evidence has recently revealed that S1P and FTY720 might have intracellular effects that are not mediated by cell surface receptors (Pfeilschifter et al., 2011). They could enter in the various cells independent of the receptors, binding to cellular proteins, activating intracellular signaling pathways, and modulating cell activities through epigenetic transcriptions (Gardner et al., 2016; Hait et al., 2014; Segura‐Ulate et al., 2017). It has been shown that FTY720 increases acetylation of histone and can regulate some functions such as decreasing T cell activation, upregulation of anti‐epileptogenic effect, increasing neurotrophic factor generation, and rescuing memory deficit (Hait et al., 2009, 2014). However, the receptor‐independent activity of FTY720 has not been examined in the field of ischemic stroke (Figure 1).

## 3 EFFECT OF FTY720 ON ISCHEMIC STROKE IN EXPERIMENTAL MODELS STUDIES

Experimental stroke studies have provided strong evidence that FTY720, as an immunomodulator, play a key role in the fate of brain tissue following cerebral ischemia and has been suggested as a potential therapeutic agent for the treatment of ischemic stroke (Czech et al., 2009; Hasegawa et al., ,^2010^, ^2013^; Kraft et al., 2013; Liesz et al., 2011; Moon et al., 2015; Nazari et al., 2016; Pfeilschifter et al., 2011; Wang, Kawabori, et al., 2020; Wei et al., 2011). In this section, we describe the effects of FTY720 in various experimental stroke models and compare the use of different types of ischemic stroke models including permanent and temporary occlusion, different animal species, various therapeutic time points from acute to chronic stages, and different dose‐response effects (Table 1).

FTY720 reduces infarct size and ameliorates neurological deficit in experimental cerebral ischemia both in mice (Czech et al., 2009; Hasegawa et al., 2010; Wei et al., 2011) and rats (Hasegawa et al., 2010; Wei et al., 2011). The doses of FTY720 that administrated in the many studies were at concentrations ranging from 0.25 to 2 mg/kg (Hasegawa et al., 2010; Li et al., 2017; Wacker et al., 2009; Wei et al., 2011) that could lead to a robust lymphocytopenia (Czech et al., 2009). Its administration at the time of occlusion reduces lesion size and improves neurological function in a mouse model of cerebral ischemia by decreasing the numbers of infiltrating neutrophils, activating microglia/macrophages, and reducing pro‐apoptotic processes such as nuclear translocation of apoptosis‐inducing factor (AIF) in the ischemic lesion (Czech et al., 2009). Wei et al. also suggested the FTY720 might protect endothelial cells through decreasing the ICAM‐1 expression and leukocyte binding and subsequent inflammation in a rat model of tMCAO (Wei et al., 2011). Furthermore, we proposed for the first time that FTY720 can prevent the synaptic plasticity impairment probably via postsynaptic mechanism and improve memory impairment in MCAO rats (Nazari et al., 2016).

In addition to the anti‐inflammatory effects of FTY720 via lymphopenia, there is growing evidence that showed the preservation of the BBB and direct neuroprotective were responsible for neurological recovery in ischemic stroke models. Recently results showed that FTY720 has protective effects against major vascular disruption in the brain after ischemia/reperfusion via the S1P signaling pathway, independent of its effects on lymphocytes. FTY720‐induced attenuation of ischemia‐induced β‐catenin degradation suggests that its effect is related to the maintenance of vascular integrity after severe ischemia‐reperfusion (Salas‐Perdomo et al., 2019) (Table 1). Administration of FTY720 immediately after reperfusion, reduces infarct volume and improves neurological score at 24 and 72 hr after middle cerebral artery occlusion in rats (Hasegawa et al., 2010). Concerning the signaling pathway mediating the protective effect of FTY720, it was demonstrated that the neuroprotective action of FTY720 could be imitated by the selective S1P1 agonist SEW2871 and inhibited by the S1P1/S1P3‐selective antagonist VPC23019, suggesting an S1P1‐mediated effect. The neuroprotective and anti‐apoptotic effect of FTY720 is associated with deactivation of caspase‐3, activation of Akt/ERK, and Bcl‐2 upregulation through S1P1 receptor activation (Hasegawa et al., 2010). This assumption was supported by the findings that showed the protective effect of FTY720 is lost in the tMCAO model of mice lacking the SphK2 gene, but not the SphK1 gene, corroborating that the protective effect of FTY720 is mediated via phospho‐FTY720 through SphK2 (Pfeilschifter et al., 2011). It has been also shown that the expressions of S1P1, SphK1, and SphK2 were significantly decreased after tMCAO and FTY720 could reduce infarct volume in the cortex, but not the subcortex, and improve sensorimotor function after 24 hr of MCAO in rats through act upon S1P1 on neurons (Hasegawa et al., 2013; Pfeilschifter et al., 2011). It has been indicated that hypoxic preconditioning (HPC)‐induced ischemic tolerance is mediated by an increase in microvascular SphK2 activity that could ultimately lead to S1P‐mediated alterations in gene expression. So, this pathway may be a novel endogenous pathway for ischemic protection that could be targeted for the treatment of stroke (Wacker et al., 2009). The direct neuroprotective ability of FTY720 has been also revealed in cortical ischemic stroke (Li et al., 2017; Shang et al., 2020) and white matter ischemic damage induced by chronic hypoperfusion (Qin et al., 2017). Two meta‐analyses have also supported the efficacy of FTY720 in animal models of stroke and its use as a treatment in stroke patients (Dang et al., 2021; Liu et al., 2013. There are conflicting results among the studies that evaluated the protective action of FTY720 against ischemic stroke. For example, Liesz et al. did not find any beneficial effect of FTY720 in the outcome of permanent murine cerebral ischemia, despite effective lymphopenia and reduction of post‐ischemic brain lymphocyte influx (Liesz et al., 2011) (Table 1).

## 4 FTY720 EFFECTS ON ISCHEMIC STROKE IN CLINICAL TRIALS

The sudden and extensive influx of lymphocytes from the periphery to the ischemic region induces focal inflammatory responses that lead to tissue death and worsens clinical outcomes in patients with acute ischemic stroke. A clinical pilot trial in 22 patients with acute and anterior cerebral circulation occlusion stroke showed that oral administration of FTY720 for 3 consecutive days reduced lymphocyte migration to the injury site during the first 72 hr of ischemic stroke. It also led to a significant reduction of secondary lesion enlargement, microvascular permeability, attenuation of hemorrhagic transformation, and promotion of neurological recovery (Fu et al., 2014). It has been found that patients who receive the combination of FTY720 with alteplase for 3 consecutive days within 4.5 hr of the onset of ischemic stroke, exhibit lower circulating lymphocytes, smaller lesion volumes, less hemorrhagic transformation, and better clinical outcomes compared with patients who received alteplase alone (Zhu et al., 2015). Altogether, the clinical findings support research results that FTY720 could be considered as a useful therapy for ischemic stroke. Although beneficial effects of FTY720 have been known in the treatment of patients with ischemic stroke, it also has important side effects. FTY720 affects the cardiovascular system and induces cardiac arrhythmias, increases blood pressure, and can also lead to bradycardia and atrioventricular blockages (Racca et al., 2016). Furthermore, FTY720‐induced lymphocytopenia can enhance stroke‐related immunodepression that has been identified as an essential cause of ‐post‐stroke infection and mortality (Klehmet et al., 2009; Meisel et al., 2005). It also can increase the risks of teratogenicity in pregnant women taking it before or during pregnancy (Karlsson et al., 2014). Therefore, FTY720 should be cautiously administrated to patients with current or previous heart failure and pregnant women.

## 5 CONCLUSION

In this review, the mechanisms associated with the neuroprotective effects of FTY720 were discussed. We also reviewed the experimental and clinical data, suggesting the beneficial role of FTY720 in preventing the progression of brain injury following cerebral ischemic stroke via three mechanizes including functional antagonistic mechanism, which is mediated by its lymphocyte homing action, functional agonist mechanism which is known as direct neuroprotective effects of FTY720, and receptor‐independent mechanisms. In summary, FTY720 seems to be a candidate drug for therapeutic intervention in ischemic stroke. However, further studies are required to confirm FTY720 as a prescription drug for ischemic stroke patients.

## CONFLICT OF INTEREST

The authors declare no conflict of interest and declare that the research was conducted in the absence of any commercial or financial relationships that could be construed as a potential conflict of interest.

## AUTHOR CONTRIBUTIONS

All authors have authorship criteria, contributed significantly, and in agreement with the content of the manuscript.

## ETHICAL APPROVAL

This manuscript is in accordance with the authorship statement of ethical standards for a manuscript submitted to Brain and behavior, and has not also been published previously and is not under consideration at another journal.

### PEER REVIEW

The peer review history for this article is available at https://publons.com/publon/10.1002/brb3.2179.
